# Supplementary material for: Immune Checkpoint Inhibitor Therapy and Associations with Clonal Hematopoiesis
Source: Int J Mol Sci. 2024 Oct 15;25(20):11049. doi: 10.3390/ijms252011049 (PMC11508050; doi:10.3390/ijms252011049)
Supplement: Supplementary file 1 [file ijms-25-11049-s001.zip › ijms-3220574-supplementary.pdf]

Supplementary files for "Immune Checkpoint Inhibitor Therapy and Associations with Clonal Hematopoiesis" (Singh, A et al)

Supplemental Figure S1: Serial blood sample analysis for somatic mutations, following ICB exposures in patients with melanoma
Supplemental Figure S2: The characteristics of patients whose serial samples were available is shown in supple-mental Figures S2a (MEL) and S2b (NSCLC).

Supplemental Figure S1

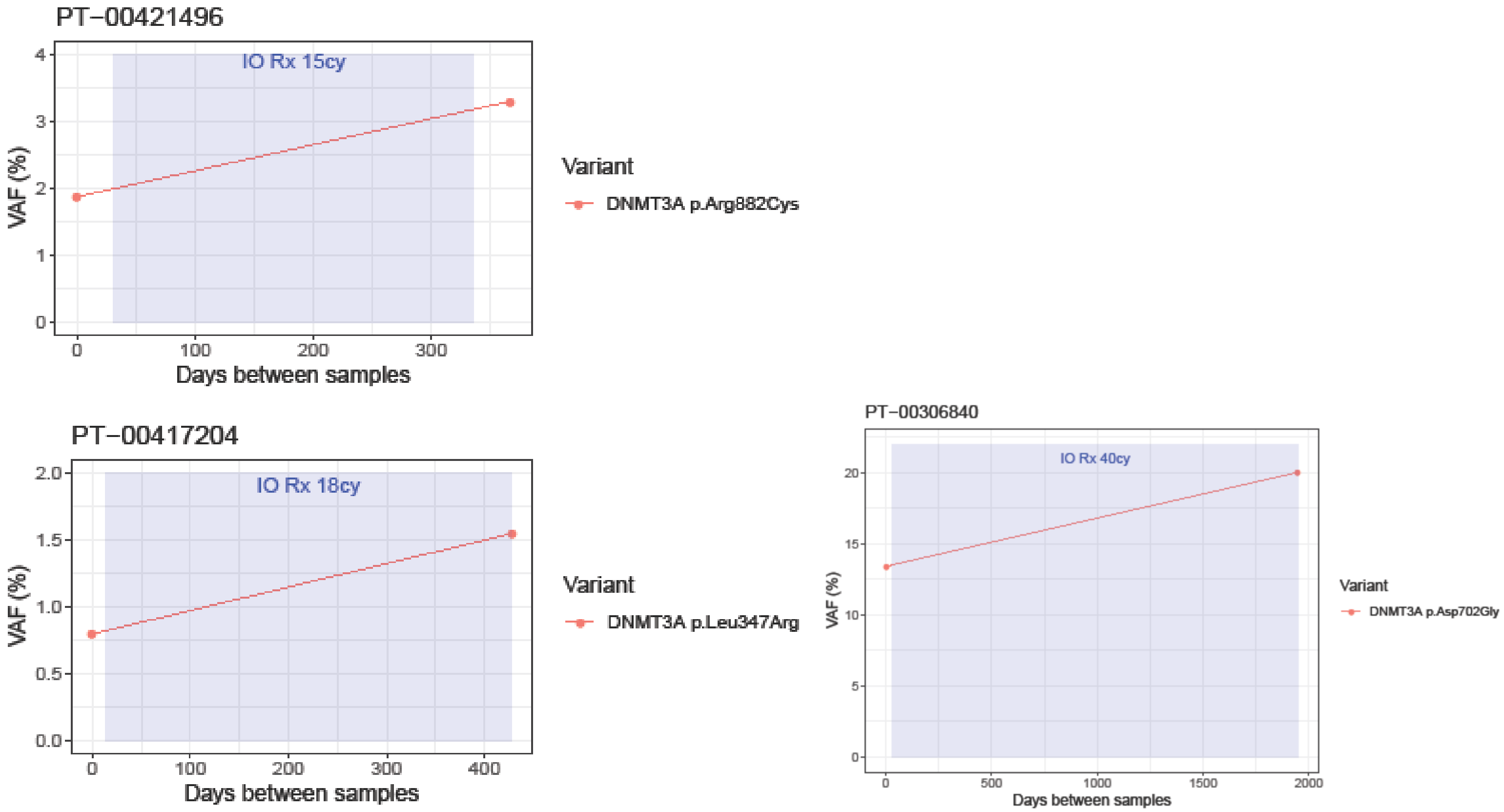
PT−00421496
IO Rx 15cy
VAF (%)
Days between samples
Variant
DNMT3A p.Arg882Cys
PT−00417204
IO Rx 18cy
VAF (%)
Days between samples
Variant
DNMT3A p.Leu347Arg
PT−00306840
IO Rx 40cy
VAF (%)
Days between samples
Variant
DNMT3A p.Asp702Gly

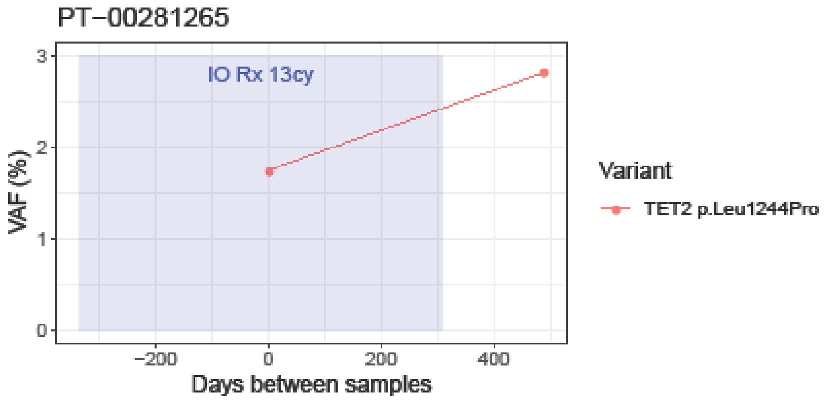
PT−00281265
IO Rx 13cy
VAF (%)
Days between samples
Variant
TET2 p.Leu1244Pro

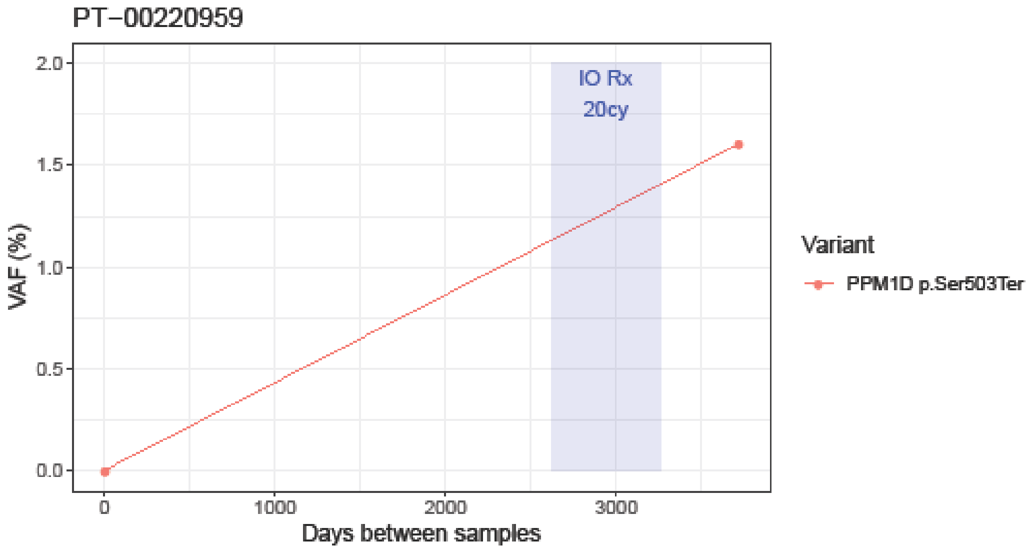
PT−00220959
IO Rx
20cy
VAF (%)
Days between samples
Variant
PPM1D p.Ser503Ter

Supplemental Figure S2a:

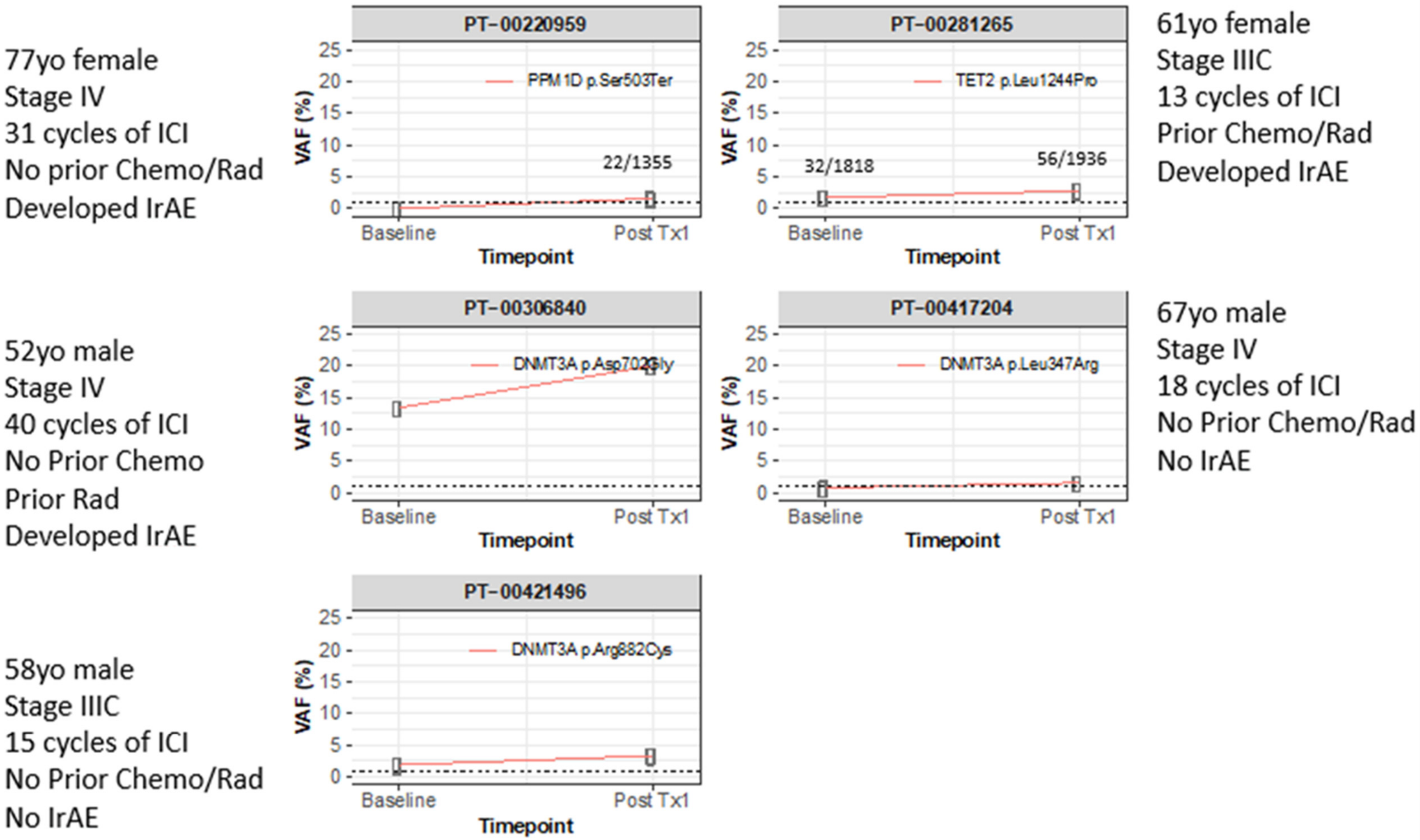

Supplemental Figure S2b

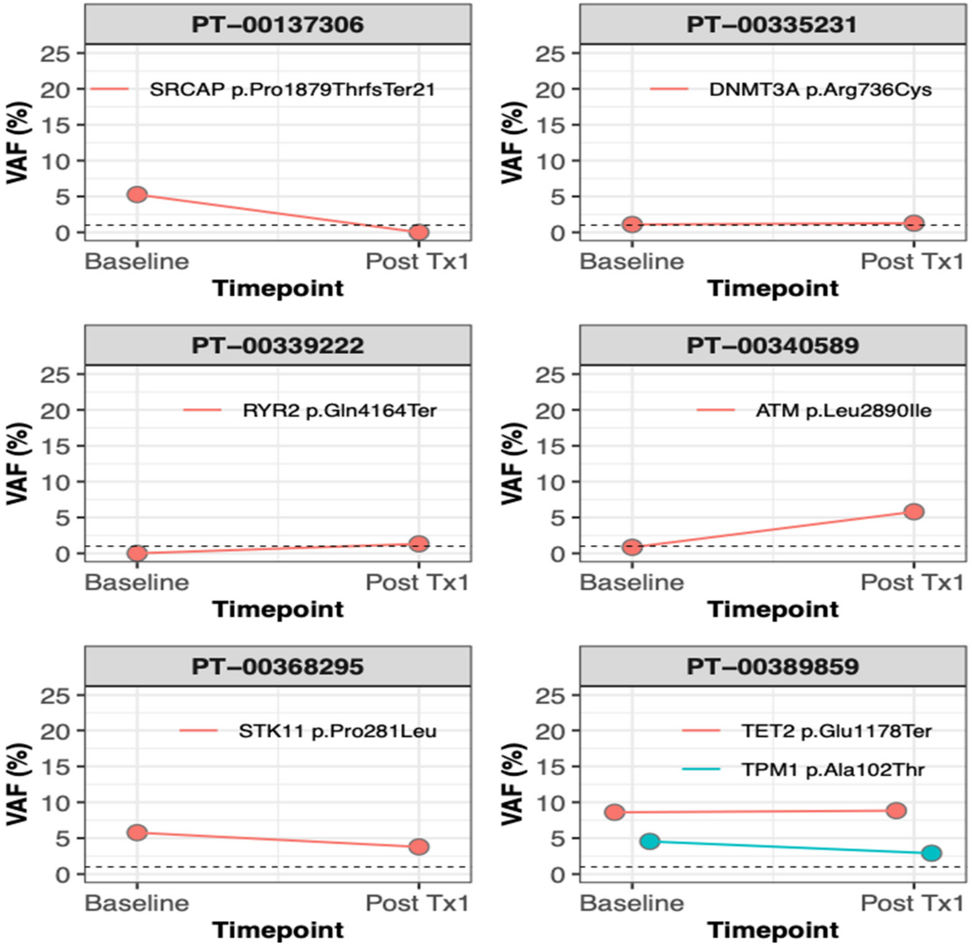

61yo female
Non-Metastatic Adeno
7cy ICI (5mo)
Prior Chemo/Rad

61yo female
Metastatic Adeno
6cy ICI (10mo)
Prior Chemo/Rad

77yo female
Metastatic SCC
18cy ICI (24mo)

66yo female
Non-Metastatic SCC
42cy ICI (27mo)
Prior Chemo/Rad

78yo female
Metastatic Adeno
17cy ICI (16mo)
Prior Chemo/Rad

72yo female
Metastatic Adeno
25cy ICI (20mo)
No Prior Chemo
Prior Rad

Supplemental Table S1: Comparison of characteristics in melanoma patients with clonal hematopoiesis (CH) vs without CH

	All	CH-	DNMT3A CH+	non-DNMT3A CH+
**Parameter**	**N = 31**	**n = 24**	**n = 5**	**n = 2**
Age	62.5 (33-88)	60.7 (33-77)	67.8 (52-88)	64 (61-67)
ANC	4.4 (1.9-7.6)	4.4 (1.9-6.3)	4.3 (2-7.6)	5.7 (3.8-7.6)
Platelets	237.8 (134-531)	233.4 (134-408)	199 (149-244)	392 (253-531)
Hgb	14 (8.4-16.6)	14.4 (11.6-16.6)	13.6 (11.2-16)	11.2 (8.4-14.1)
MCV	91.6 (82-102)	91.5 (84-102)	92 (84-101)	88.5 (82-95)
RDW	13.5 (12-17.7)	13.2 (12-16.3)	14.8 (12.8-17.7)	14.1 (13.6-14.6)
Immunotherapy_cycles	22 (1-71)	23.8 (3-71)	21 (10-40)	7 (1-13)

Supplemental Table S2 : Change in variant allele frequency (VAF2 vs pre-exposure VAF1) of somatic mutations with exposure to ICB in patients with melanoma

Patient ID	Gene	HGVSp	VAF1	VAF2
PT-00220959	PPM1D	ENSP00000306682.2:p.Ser503Ter	0	0.016
PT-00281265	TET2	ENSP00000442788.1:p.Leu1244Pro	0.0174	0.0281
PT-00306840	DNMT3A	ENSP00000264709.3:p.Asp702Gly	0.1339	0.2001
PT-00417204	DNMT3A	ENSP00000264709.3:p.Leu347Arg	0.0079	0.0154
PT-00421496	DNMT3A	ENSP00000264709.3:p.Arg882Cys	0.0186	0.0329

**Supplemental Table S3: Clonal hematopoiesis genes list**<br>We performed targeted- sequencing of 93 myeloid neoplasm specific genes.
ACTA2, ACTC1, ANGPTL4, ANKRD26, APC, APOA5, APOB, APOC3, ASXL1, ATM, BAP1, BARD1, BMPR1A, BRAF, BRCA1, BRCA2, BRIP1, CBL, CDH1, CDK4, CDKN2A, CEBPA, CHEK2, COL3A1, DDX41, DNMT3A, DSC2, DSG2, DSP, EPCAM, ETV6, FBN1, FLT3, GATA1, GATA2, GLA, GREM1, IDH1, IDH2, JAK2, KCNH2, KCNQ1, KDM1A, KRAS, LDLR, LMNA, LPA, LPL, MITF, MLH1, MPL, MSH2, MSH6, MUTYH, MYBPC3, MYH11, MYH7, MYL2, MYL3, NBN, NPC1L1, NPM1, NRAS, PALB2, PCSK9, PKP2, PMS2, POLD1, POLE, PPM1D, PRKAG2, PTEN, RAD51C, RAD51D, RUNX1, RYR2, SCN5A, SF3B1, SMAD3, SMAD4, SRP72, SRSF2, STK11, TET2, TGFBR1, TGFBR2, TMEM43, TNNI3, TNNT2, TP53, TPM1, U2AF1, ZRSR2
